# Periostin enhances adipose-derived stem cell adhesion, migration, and therapeutic efficiency in Apo E deficient mice with hind limb ischemia

**DOI:** 10.1186/s13287-015-0126-x

**Published:** 2015-07-24

**Authors:** Jinbao Qin, Fukang Yuan, Zhiyou Peng, Kaichuang Ye, Xinrui Yang, Lijia Huang, Mier Jiang, Xinwu Lu

**Affiliations:** Department of Vascular Surgery, Shanghai Ninth People’s Hospital Affiliated to Shanghai JiaoTong University, School of Medicine, Shanghai, 200011 People’s Republic of China; Vascular Center of Shanghai JiaoTong University, Shanghai, 200011 People’s Republic of China

## Abstract

**Introduction:**

Therapeutic angiogenesis by transplantation of autologous/allogeneic adipose-derived stem cells (ADSCs) is a potential approach for severe ischemic diseases. However, poor viability, adhesion, migration and differentiation limit the therapeutic efficiency after the cells were transplanted into the targeted area. Periostin, an extracellular matrix protein, exhibits a critical role in wound repair as well as promotes cell adhesion, survival, and angiogenesis.

**Method:**

ADSCs were obtained and genetically engineered with periostin gene (P-ADSCs). The viability, proliferation, migration, and apoptosis of P-ADSCs under hypoxia were analyzed. Moreover, P-ADSCs were implanted into Apo E deficient mice with hind limb ischemia. The Laser Doppler perfusion index, immunofluorescence, and histological pathology assay were tested to evaluate the therapeutic effects. The associated molecular mechanism of periostin on the proliferation, adhesion, migration, and differentiation of ADSCs was also analyzed.

**Results:**

The in vitro studies have shown that periostin-transfected ADSCs (P-ADSCs) promoted viability, proliferation, and migration of ADSCs. Apoptosis of ADSCs was inhibited under hypoxic conditions. The Laser Doppler perfusion index was significantly higher in the P-ADSCs group compared with that in the ADSC and control groups after 4 weeks. Immunofluorescence and histological pathology assay showed that the P-ADSCs were in and around the ischemic sites, and some cells differentiated into capillaries and endothelium. Microvessel densities were significantly improved in P-ADSCs group compared with those in the control group. The molecular mechanisms that provide the beneficial effects of periostin were connected with the upregulated expression of integrinβ1/FAK/PI3K/Akt/eNOS signal pathway and the increased secretion of growth factors.

**Conclusion:**

Overexpression of periostin by gene transfection on ADSCs promotes survival, migration, and therapeutic efficiency, which will bring new insights into the treatment of critical limb ischemia.

**Electronic supplementary material:**

The online version of this article (doi:10.1186/s13287-015-0126-x) contains supplementary material, which is available to authorized users.

## Introduction

Adipose-derived stem cells (ADSCs) are suitable seed cells for regenerative medicine because of their abundant sources, easy isolation and expandability, and multipotency [1]. Previous studies have shown the therapeutic potential of ADSCs in repairing myocardial infarctions, hind limb ischemia, and stroke [2–4]. However, poor viability, adhesion, migration, and differentiation of transplanted cells limit the therapeutic efficiency when the stem cells are transplanted into the targeted area or organs [5, 6].

Several studies have documented that the leading causes of cell death are scarcity in nutrients and oxygen, especially under ischemic environment conditions. The lack of vital nutrients results in loss of survival, adhesion, migration, and differentiation signals from inadequate interaction between cells and the microenvironment around the extracellular matrix [5–7]. To acquire effective therapeutic modalities, enhancing the ability of stem cells to endure the rigors of the ischemic microenvironment, the inflammatory responses, and the stimulation of apoptosis factors is crucial. Several attempts have been performed to enhance the therapeutic effects of stem cells by modification of cells with special genes, growth factors, and cytokines [8–10].

As one of the extracellular matrix proteins (ECM), periostin, a 90 kDa secreted cell adhesion protein, is highly expressed in fibrous connective tissues, such as bones, heart valves, skin, tendons, and periodontal ligaments [11–13]. Periostin is also prominent in neoplastic tissues and exhibits an important function in tumor growth, survival, angiogenesis, and epithelial–mesenchymal transition [14, 15]. Periostin is also highly expressed in tissue trauma or wound repair, and participates in repairing tissue damage [16–18]. Periostin is remarkably expressed during tissue physiology, wound repair, and triggered remodeling of pathophysiological ECM, which includes the regeneration of cardiomyocytes after myocardial infarction [13, 19], the epithelial–mesenchymal transition in neoplastic tissues [20, 21], and the remodeling of pulmonary vasculature [22, 23]. We therefore hypothesized that the overexpression of periostin by gene transfection on ADSCs promotes survival, migration, and multidifferentiation of cells under ischemic conditions.

In this study, ADSCs were genetically engineered with the periostin gene (P-ADSCs). The viability, proliferation, migration, and apoptosis of P-ADSCs under hypoxia were analyzed. Moreover, P-ADSCs were implanted into apolipoprotein E (Apo E)-deficient mice with hind limb ischemia. The laser Doppler perfusion index, immunofluorescence, and a histological pathology assay were tested to evaluate the therapeutic effects. We also analyzed the associated molecular mechanism of periostin on the proliferation, adhesion, migration, and differentiation of ADSCs.

## Materials and methods

### Isolation and characterization of ADSCs

ADSCs and green fluorescent protein (GFP)-ADSCs were isolated from 4-week-old C57/BL6 or C57/BL6-GFP mice (Shanghai SLAC Laboratory Animal Co. Ltd, Shanghai, China) inguinal adipose tissues and expanded as described previously [24, 25]. All animal procedures were performed in accordance with the Guidelines of the Animal Experiment and Care Committee of Shanghai JiaoTong University School of Medicine.

The characteristics of GFP-ADSCs were analyzed by flow cytometry. Briefly, passage 3 (P3) and passage 6 (P6) GFP-ADSCs were harvested and washed with phosphate-buffered solution (PBS). The cells were then incubated with phycoerythrin (PE)-conjugated anti-mouse antibodies against CD11b, CD31, CD34, CD44, CD45, CD105, CD133, major histocompatibility complex (MHC)-II, CD90, and Sca-1 for 30 minutes at 4 °C in the dark. Normal rabbit IgG antibodies were used as negative isotype control groups (eBioscience, San Diego, CA, USA). The cells were resuspended in ice-cold PBS and analyzed via flow cytometry (Beckman Coulter, Fullerton, CA, USA).

### Gene transfection of ADSCs

We used a lentiviral vector for gene transfection to ensure stable gene transfection of ADSCs. Full-length mouse periostin cDNA was cloned into the pLenti6.3-MCS shuttle vector (Invitrogen, Carlsbad, CA, USA) as described previously [26, 27]. Lentiviruses were produced by transfecting 293T cells. ADSCs were incubated with the virus and polybrene for 24 hours for transduction. The periostin cDNA was amplified using the following primer set: forward, 5′-gccagatctcttcctggacggagctcagggctgaag-3′; and reverse, 5′-aaacccagaggccagaccacagagtttatataatcctaaatcaacgat-3′. Nontransfected P-ADSCs and cells transfected with empty pLenti6.3-MCS shuttle vector served as control. Immunofluorescent staining and western blot were used to assess the expression of periostin in ADSCs transduced with empty vector and periostin as described previously [27]. Small interfering RNA (siRNA) targeting different sequences (angiopoietin and transforming growth factor beta) in the periostin gene were purchased from and synthesized by R&S Co., Ltd (Shanghai, China). ADSCs were transfected with 50 nM siRNA mixed with 5 ml Lipofectamine 2000 (Invitrogen) according to the manufacturer’s protocol. Nontargeting siRNA and the stealth RNA negative control were used as controls.

### Cell viability assay

The viability of P-ADSCs and ADSCs was evaluated using the cell counting kit-8 (CCK-8; Dojindo Laboratories, Kumamoto, Japan) following the manufacturer’s instructions as described previously [24, 25]. Briefly, ADSCs and P-ADSCs were trypsinized and cultured in 96-well plates at 5 × 10^3^ cells/well at 37 °C and 5 % CO_2_. After 24 hours of incubation, 10 μl CCK-8 in 100 μl Dulbecco’s modified Eagle’s medium (DMEM; Hyclone, Ottawa, Canada) were added per well and incubated for 2 hours, and unattached cells were removed with PBS. The absorbance optical density (OD) values were measured at 450 nm using a MRX Microplate Reader (Dynex Technologies, VA, New York, USA). Cell growth was measured every day from days 1 to 6, and each assay was performed in quadruplicate. To test the effect of periostin on ADSCs, cells were treated with different concentrations of periostin (0, 12.5, 25, 50, and 100 ng/ml) for 24 hours. The viability of ADSCs was evaluated using CCK-8 following the manufacturer’s instructions.

### Effect of periostin on ADSC apoptosis under hypoxia

ADSCs and P-ADSCs were cultured at 80 % confluency, and then incubated in hypoxic conditions with a gas mixture of 10 % CO_2_, 5 % H_2_, and 85 % N_2_ for 24 hours as described previously [27, 28]. The apoptotic rate was measured using the Annexin V apoptosis detection kit (BD Bioscience, New York, USA) as described previously [24]. The cells were pelleted and analyzed by flow cytometry (Beckman Coulter). The green fluorescence emitted by GFP and the red fluorescence of Annexin V were measured using 488 nm and 555 nm bandpass filters, respectively. A total of 1 × 10^5^ cells were analyzed in each sample.

For western blotting analysis, P-ADSC and ADSC lysates were collected at the termination of the experiment. The protein samples were subjected to SDS-PAGE/immunoblotting analysis using anti-Bcl-2 antibody, anti-Bax antibody, and anti-β-actin antibody (1:500; Abcam, Cambridge, UK). The anti-apoptotic gene *Bcl-2* and the apoptotic gene *Bax* were also analyzed using quantitative real-time (qRT) PCR following the manufacturer's instructions as described previously [24]. PCR with cDNA was performed using the following primers: Bcl-2 forward, 5′-CGG GAG AAC AGG GTA TGA TAA-3′ and reverse, 5′-TCA GGC TGG AAG GAG AAG AT-3′; and Bax forward, 5′-GGC AGA CAG TGA CCA TCT TTG-3′ and reverse, 5′-ATT CAT CCC AGG AAA ATG TCA TA-3′.

### Assays for cell adhesion and migration

To explore the effect of periostin on the adhesion ability of ADSCs, the ADSCs, P-ADSCs, and siRNA were plated at a density of 1 × 10^5^ cells/well in a six-well plate and were incubated for 30 minutes at 37 °C. The wells were washed three times, and the number of attached cells was estimated by microscopic cell counting using a hemacytometer. Each experiment was performed in triplicate wells and repeated at least three times.

Migration was assayed by a modified Boyden chamber method using a Boyden chamber and an 8 μm polyethylene terephthalate (PET) membrane (R&D Systems Inc., Minneapolis, MN, USA). ADSCs were trypsinized and suspended at a concentration of 2 × 10^4^ cells/ml in DMEM supplemented with 0.5% fetal bovine serum (FBS). The ADSC suspension (100 μl) was placed in the upper chamber, and 600 μl DMEM containing 25 ng/ml periostin was placed in the lower chamber. The chamber was incubated at 37 °C and 5 % CO_2_ for 12 hours. The filter was removed, and the cells on the upper side of the filter were scraped off with a cotton tip. The ADSCs that migrated to the lower side of the filter were fixed in 4 % paraformaldehyde and stained with 4′,6-diamidino-2-phenylindole (DAPI; DAKO, USA). Experiments were performed in triplicate.

For western blotting analysis, P-ADSC, ADSC, and siRNA lysates were collected at the termination of the experiment. The protein samples were subjected to SDS-PAGE/immunoblotting analysis using anti-integrin-β1 antibody, anti-FAK and P-FAK antibody, anti-PI3K antibody, anti-Akt and P-AKT antibody, anti-eNOS and P-eNOS antibody, and anti-β-actin antibody (1:500; all from Abcam). The relative integrated density of the protein bands was quantified using the Odyssey infrared imaging system (LI-COR, Lincoln, NE, USA).

### Induction of hind limb ischemia and cell transplantation

All animal protocols were approved by the Animal Experiment and Care Committee of Shanghai JiaoTong University School of Medicine. Male Apo E-deficient mice (6–8 weeks old, *n* = 24; Shanghai Research Center for Model Organism, China) with a mean age of 10 ± 1.6 months were fed a high-cholesterol diet (containing 21% fat, 0.15% cholesterol) for at least 6 months before the experiment. This animal model is generally accepted as the atherosclerosis AS mouse model, and the strain for the Apo E-deficient mice is also C57BL/6. These mice can imitate pathological changes in atherosclerotic lesions (large necrotic core, thin fibrous cap, and abundant macrophages) with high consistency to human atherosclerosis, when fed with a high-cholesterol diet. Ischemia of the right hind limb was induced in male Apo E-deficient mice as described previously [24]. The animals were then anesthetized with pentobarbital sodium (0.5 mg/g). The proximal portion of the femoral artery, including the superficial and deep branches, was cut and ligated twice using 8–0 silk sutures, and the overlying skin was closed. After 24 hours, the cells were suspended in 100 μl serum-free medium (1 × 10^6^ cells/ml) and injected into tissues from the injured region at three different sites (gastrocnemius, gracilis, and quadriceps muscles) in the ischemic leg. The mice were randomly divided into four groups: control group (*n* = 6) that received DMEM, the ADSC group (*n* = 6), the P-ADSC group (*n* = 6), and the sham group (*n* = 6). A laser Doppler blood flow meter (Omegaflow floci; Omegawave, Tekniikantie, Finland) was used to measure the cutaneous blood flow 1, 2, and 4 weeks after transplantation.

### Immunofluorescence and immunohistochemistry staining

The animals were anesthetized, and perfusion fixation was performed to investigate the survival of P-ADSCs, and the possibility of differentiation into endothelial cells or capillaries in ischemic muscles. The muscles were then excised for histological and immunohistochemical examination after 4 weeks. The frozen sections were subjected to immunofluorescent, hematoxylin and eosin (HE), and immunohistochemistry staining following the manufacturer’s instructions. Muscle sections were stained with CD31 (PECAM-1; Abcam) and α-smooth muscle actin (Epitomics; Abcam), and the nuclei were stained with DAPI (Dako) as reported previously [24].

### Measurement of paracrine factors by enzyme-linked immunosorbent assay

The levels of basic fibroblast growth factor (bFGF), hepatocyte growth factor (HGF), vascular endothelial growth factor (VEGF), and platelet-derived growth factor (PDGF) released from ADSCs into the culture medium at 48 hours were directly measured by enzyme-linked innunosorbent assay (ELISA) kit according to the manufacturer's instructions (R&D Systems Inc.). The basal medium was used as control. Each experiment was performed in triplicate wells and repeated at least three times.

### Statistical analysis

Data are presented as mean values ± standard deviation (SD). For quantitative comparison and analysis, the values were subjected to a Student’s *t* test and one-way analysis of variance. All experiments were repeated at least three times. *P* <0.05 (*) was considered statistically significant.

## Results

### Characterization and flow cytometry analysis of ADSCs

**Fig. 1:**
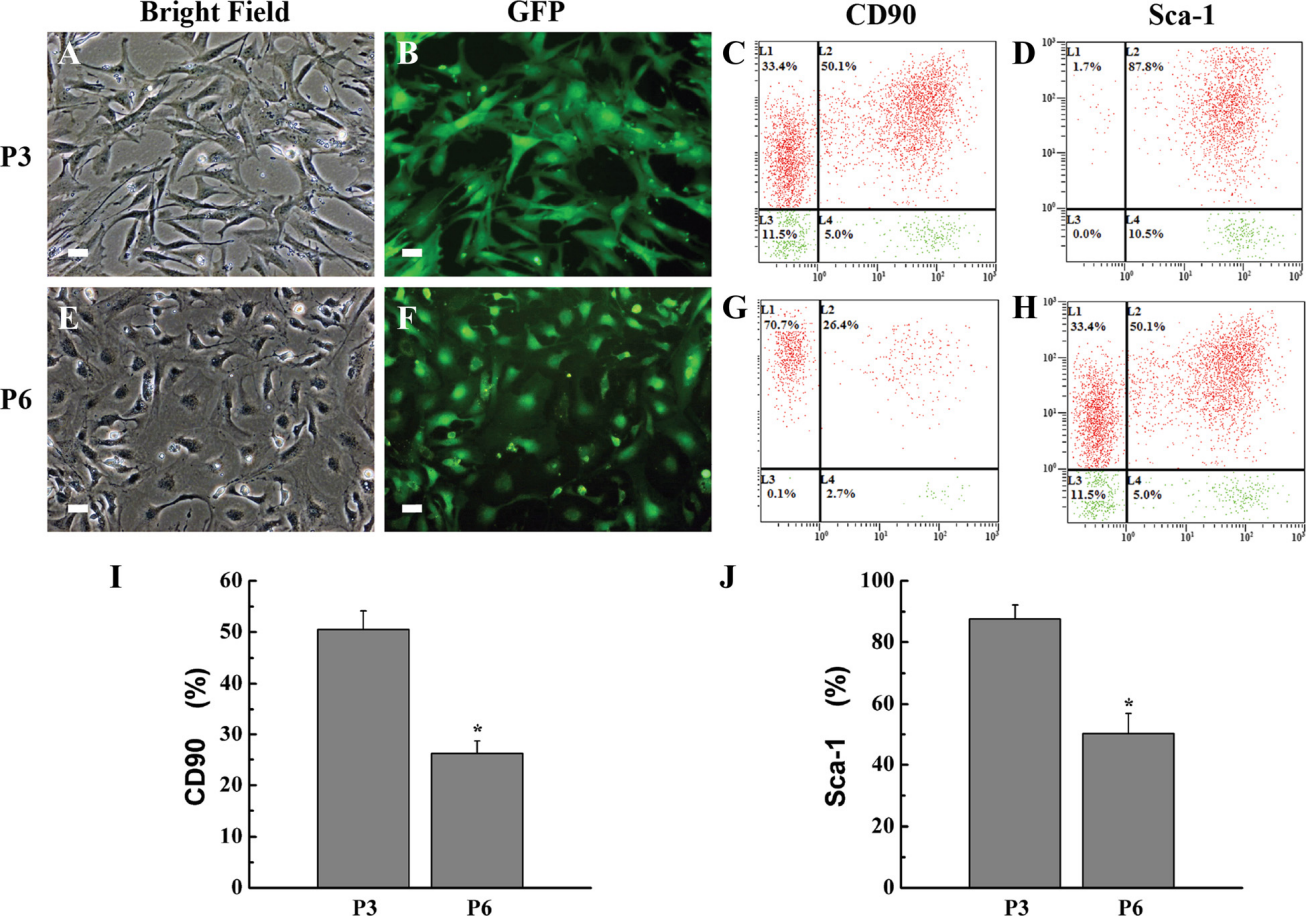
Characterization and flow cytometry analysis of ADSCs. P3 GFP-ADSCs adopted a more uniform fibroblast-like, spindle-shaped population **a**, and GFP was observed under fluorescence microscopy **b**. P6 GFP-ADSCs adopted increased aging or dedifferentiated shape with irregularity **e**. The intensity of GFP gradually reduced under fluorescence microscopy **f**. Flow cytometry revealed that CD90 and Sca-1 surface antigens of P3 **c, d** and P6 **g, h** GFP-ADSCs were gradually reduced with statistically significant differences (*P <0.05) **i, j**. Scale bar = 100 μm. *GFP* green fluorescent protein, *P3* passage 3, *P6* passage 6

ADSCs and GFP-ADSCs were isolated from inguinal adipose tissues and were quickly expanded. P3 GFP-ADSCs adopted a more uniform fibroblast-like, spindle-shaped population (Fig. 1a), and green fluorescence was also observed under fluorescence microscopy (Fig. 1b), as reported previously [24, 25]. Phenotypic analysis by flow cytometer showed that the P3 GFP-ADSCs were strongly double positive for GFP and the stem cell surface antigens CD44 (59.7 ± 2.9 %), CD105 (87.8 ± 4.2 %) (Additional file 1), CD90 (50.1 ± 3.6 %), and Sca-1 (87.5 ± 4.8 %) (Fig. 1c, d), and were negative for CD11b, CD31, CD34, CD45, CD133, and MHC-II (Additional file 1). However, P6 GFP-ADSCs adopted aging or dedifferentiation with irregularity by increasing the culture time and passages in vitro (Fig. 1e). The intensity of the green fluorescence of GFP-ADSCs was gradually reduced based on fluorescence microscopy (Fig. 1f). Flow cytometry analysis revealed that stem cell-specific marker CD90 (26.4 ± 3.1 %) and Sca-1 (51.2 ± 3.5 %) surface antigens of GFP-ADSCs (Fig. 1g, h) were gradually reduced, with statistically significant difference (*P* <0.05) (Fig. 1i, j).

### Periostin gene transfection into ADSCs

**Fig. 2:**
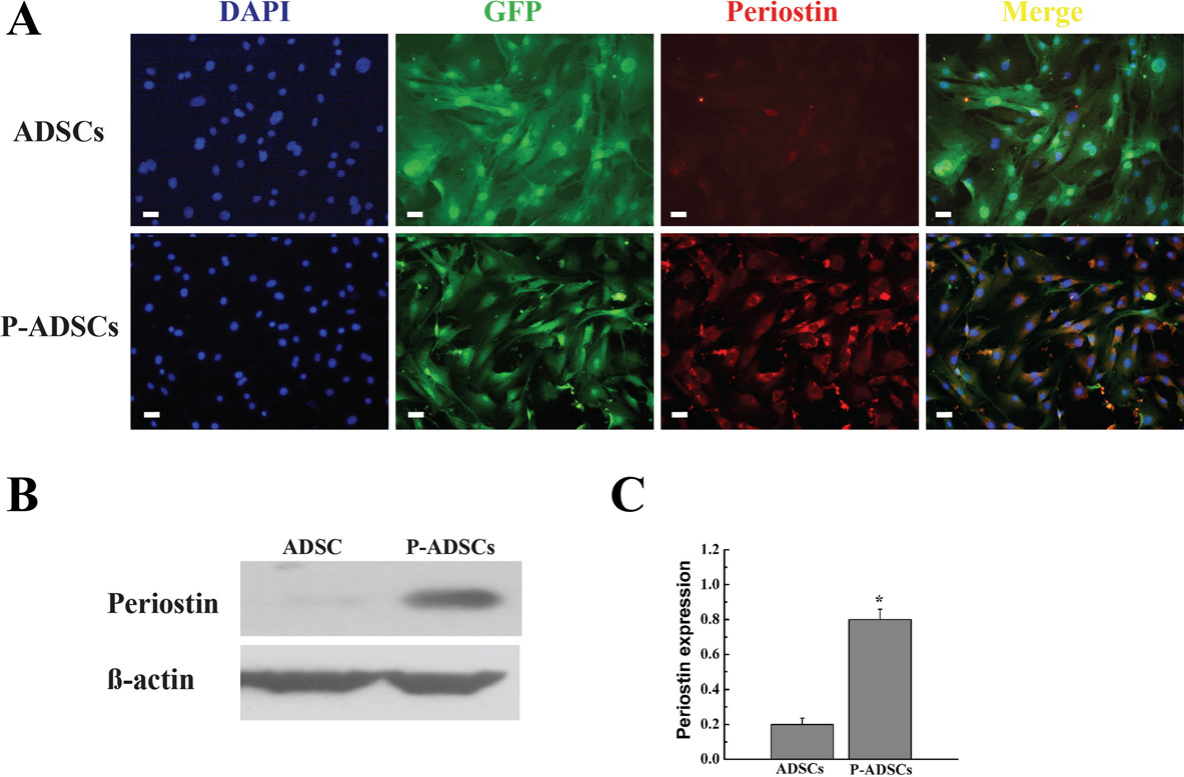
Gene transfection was adopted to overexpress periostin by ADSCs. **a** Immunofluorescence staining showed that more than 80 % of the ADSCs expressed the periostin protein. By contrast, the control ADSCs lacks the periostin protein. **b** Western blot analysis showed periostin was highly expressed in P-ADSCs but not in ADSC controls. **c** Periostin was higher in the P-ADSC group than in the control group (*P <0.05). Scale bar = 100 μm. *ADSC* adipose-derived stem cell, *DAPI* 4′,6-diamidino-2-phenylindole, *GFP* green fluorescent protein, *P-ADSC* periostin-transfected ADSC

Compared with other viral vector systems, lentivirus has many advantages, such as no significant immune response, more types of cells transfected, more stable transfected cells expressing the target genes, and the virus can be concentrated and shrunk to high titers [26, 27]. Periostin was transduced into ADSCs by lentivirus after 24 hours, and the transduction efficiency reached 80 % (Fig. 2). Insignificant difference was observed in the transduced ratio between periostin and transduced GFP cells. Immunofluorescence staining showed that more than 80 % of the ADSCs expressed the periostin markers (Fig. 2a). By contrast, the control ADSCs lacked a periostin gene marker (Fig. 2a). Western blot analyses confirmed that periostin was highly expressed in P-ADSCs but not in ADSC controls (Fig. 2b). Statistical analysis likewise revealed that the P-ADSC group exhibited significantly higher expression of periostin compared with untransfected control (*P* <0.05) (Fig. 2c). In this study, we have established a periostin gene that was efficiently transfected into the ADSC system.

### CCK-8 assay of the viability of P-ADSCs

**Fig. 3:**
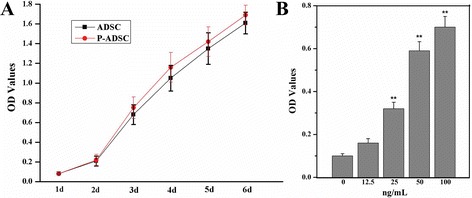
Cell viability of P-ADSCs. **a** Cells transfected with periostin clearly exhibited a higher proliferation rate compared with the untreated negative control ADSCs from 3 to 6 days (*P* <0.05, *n* = 4). **b** ADSCs were treated with different concentrations of periostin (0, 12.5, 25, 50, and 100 ng/ml) for 24 hours. **P <0.01. *ADSC* adipose-derived stem cell, *OD* optical density, *P-ADSC* periostin-transfected ADSC

To investigate the effects of periostin on the growth activity of ADSCs, cell viability was analyzed by CCK-8 assay from 1 day to 6 days (Fig. 3a). The results showed that ADSCs and P-ADSCs were initially in a stationary phase during the first day. From the second day to the 6th day, the cells underwent a logarithmic growth period. The cells transfected with periostin clearly exhibited higher proliferation rate compared with the untreated negative control ADSCs from 3 to 6 days (*P* <0.05, *n* = 4).

Different concentrations of periostin (0, 12.5, 25, 50, and 100 ng/ml) were added to test its effect on the expansion of ADSCs. As shown in Fig. 3b, the OD values of ADSCs cultured with periostin were higher compared with ADSCs cultured without periostin (*P* <0.01). With the increase in the concentration of periostin, the proliferation rate was higher. Our results demonstrate that periostin enhanced the proliferation of ADSCs.

### Effects of periostin on ADSC apoptosis under hypoxia

**Fig. 4:**
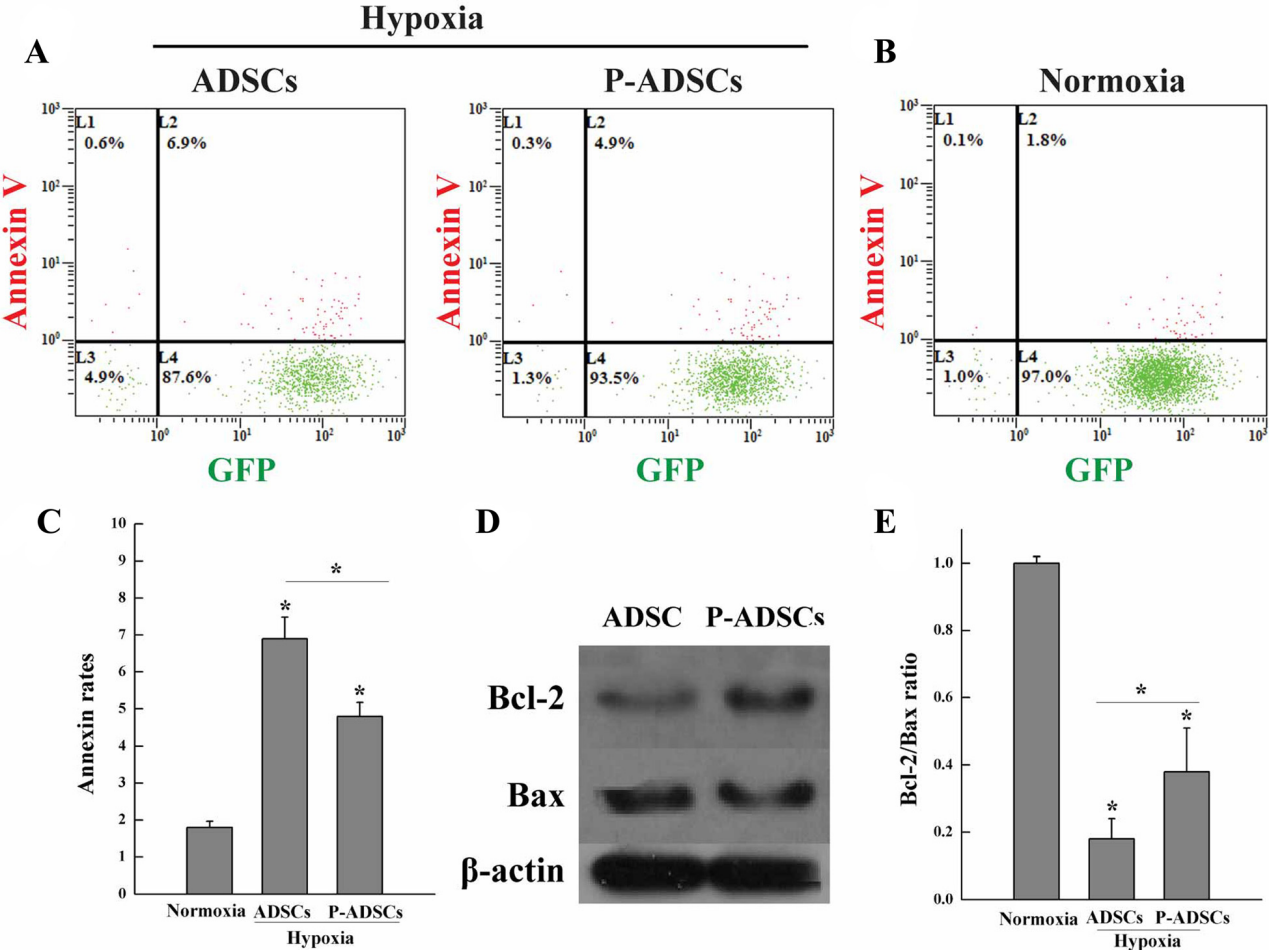
Effects of periostin on the apoptosis of ADSCs under hypoxia. Apoptosis of GFP-ADSCs transfected with or without periostin under hypoxia **a** or normoxia **b** was analyzed using flow cytometry analysis. The apoptosis rates **c** indicated that overexpression of periostin effectively enhanced the viability of ADSC. Western blotting analysis revealed that the levels of anti-apoptotic Bcl-2 increased and pro-apoptotic Bax decreased **d**, and RT-PCR **e** showed that the ratio of Bcl-2/Bax was significantly higher in P-ADSCs than in ADSCs in normoxic and hypoxic treatments. *P <0.05. *ADSC* adipose-derived stem cell, *GFP* green fluorescent protein, *P-ADSC* periostin-transfected ADSC

Several studies have revealed that hypoxic conditions affect the proliferation and apoptosis of stem cells [28, 29]. To investigate the effect of periostin on ADSC apoptosis under hypoxic conditions, the treated cells were stained with Annexin V and analyzed by flow cytometry. The apoptotic percentage of the P-ADSC group was 4.9 ± 0.18 %, compared with 6.9 ± 0.26 % for the untransfected ADSC group (Fig. 4a). Significant differences were observed between the two groups (*P* <0.05, Fig. 4c) compared with ADSCs under normoxic conditions (Fig. 4b). Western blotting analysis revealed that the levels of anti-apoptotic Bcl-2 increased and the pro-apoptotic Bax decreased significantly (Fig. 4d), and RT-PCR (Fig. 4e) showed that the ratio of Bcl-2/Bax was significantly higher in P-ADSCs than in ADSCs in normoxic and hypoxic treatments. Compared with ADSC controls under hypoxic conditions in vitro, ADSCs transfected with periostin displayed higher viability and anti-apoptotic properties, including the Bcl-2 upregulation and Bax downregulation (Fig. 4d, e). The results indicate that periostin supported cell growth factor and inhibited ADSC apoptosis under hypoxic conditions.

### Effects of periostin on adhesion and migration of ADSCs

**Fig. 5:**
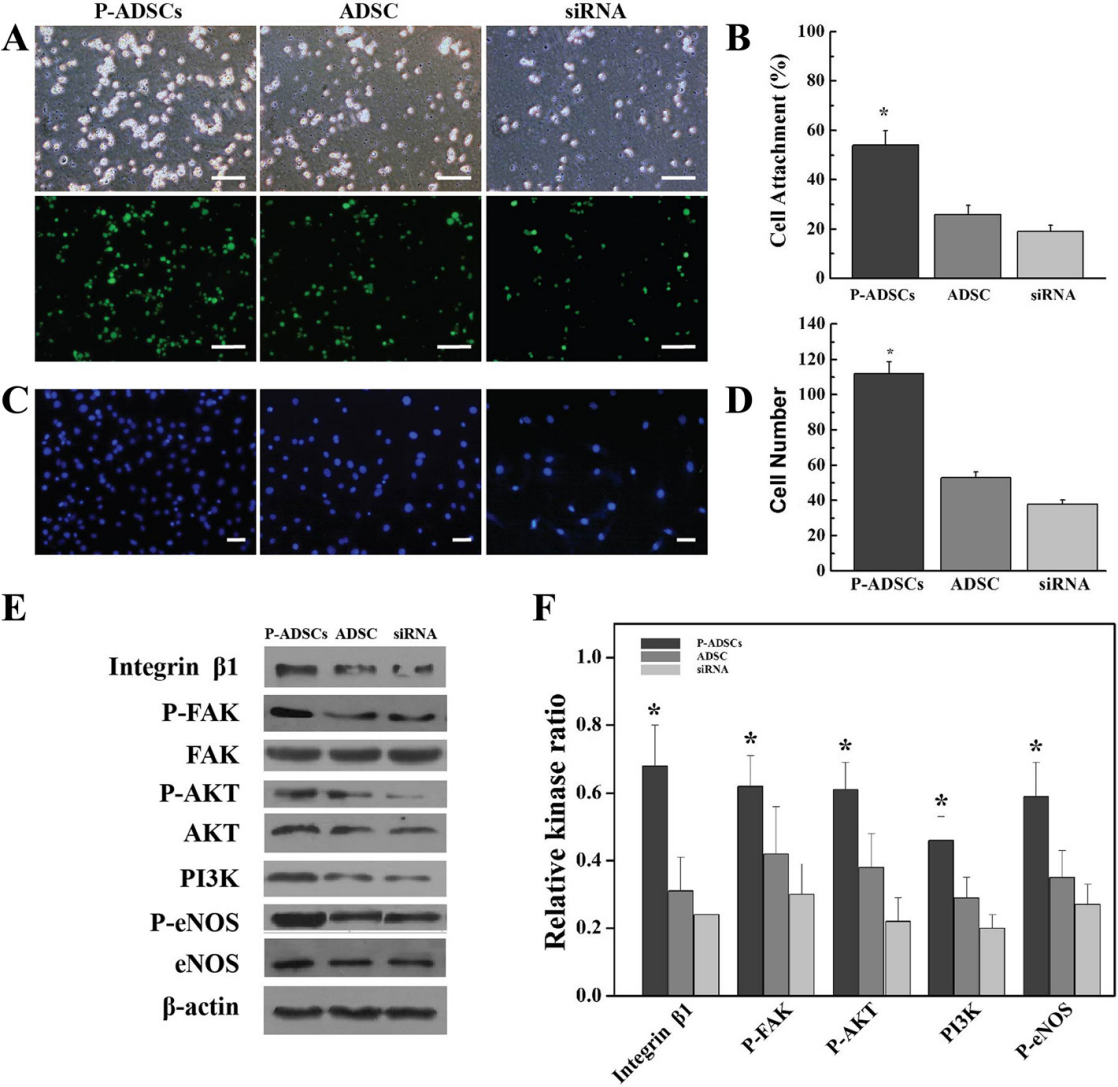
Effects of periostin on adhesion and migration of ADSCs. More attached cells were observed in the P-ADSC group compared with those in the ADSC control and siRNA groups **a**, and quantitative assays indicated that periostin overexpression effectively enhanced the adhesion of ADSCs **b**. Migration assays of the ADSCs transfected with and without periostin and siRNA groups were conducted using a Boyden chamber **c**. Data indicate that periostin overexpression improved the migration of ADSCs **d**. Scale bar = 100 μm. Western blot analysis revealed upregulation and inhibition of the integrin-β1/FAK/Akt/PI3K/eNOS signaling pathway **e**. Significant differences were observed between the P-ADSC and ADSC groups and the siRNA group **f**. *P <0.05. *ADSC* adipose-derived stem cell, *P-ADSC* periostin-transfected ADSC, *siRNA* Small interfering RNA

Following incubation for 30 minutes at 37 °C, a portion of the cells were attached to the plate of the well. The cells adopted a uniform round-shaped population, and green fluorescence was also observed under fluorescence microscopy (Fig. 5a). More cells were attached in the P-ADSC group compared with those in the ADSC control and siRNA groups. Quantitative adhesion assays of ADSCs transfected with periostin demonstrated that overexpression of periostin effectively enhanced the adhesion of ADSCs compared with those in the ADSC control and siRNA groups (Fig. 5b).

Boyden chambers were used to determine the impacts of periostin on migration of ADSCs. The results demonstrated that recombinant periostin stimulated the migration of ADSCs compared with the groups without periostin and siRNA groups (Fig. 5c). Quantitative data indicated that overexpression of periostin improved the migration of these cells (Fig. 5d).

Western blot analysis showed that the integrin-β1/FAK/PI3K/Akt/eNOS signaling pathway remained in its phosphorylated form in P-ADSCs longer than in ADSCs and siRNA groups (Fig. 5e). Quantitative assays implied that the signaling pathway was highly upregulated in P-ADSCs compared with that in the ADSC and siRNA groups (Fig. 5f). It is well documented that the activation of Akt/FAK/eNOS is critical in cell survival [30]. Akt overexpression improved neonatal cardiomyocyte graft survival. Previous reports have revealed that Akt enhanced mesenchymal stem cell survival after transplantation in myocardial infarction models. The findings suggested the significant function of periostin in regulating the adhesion and migration of ADSCs.

### Effects of P-ADSCs on blood perfusion and histology staining

**Fig. 6:**
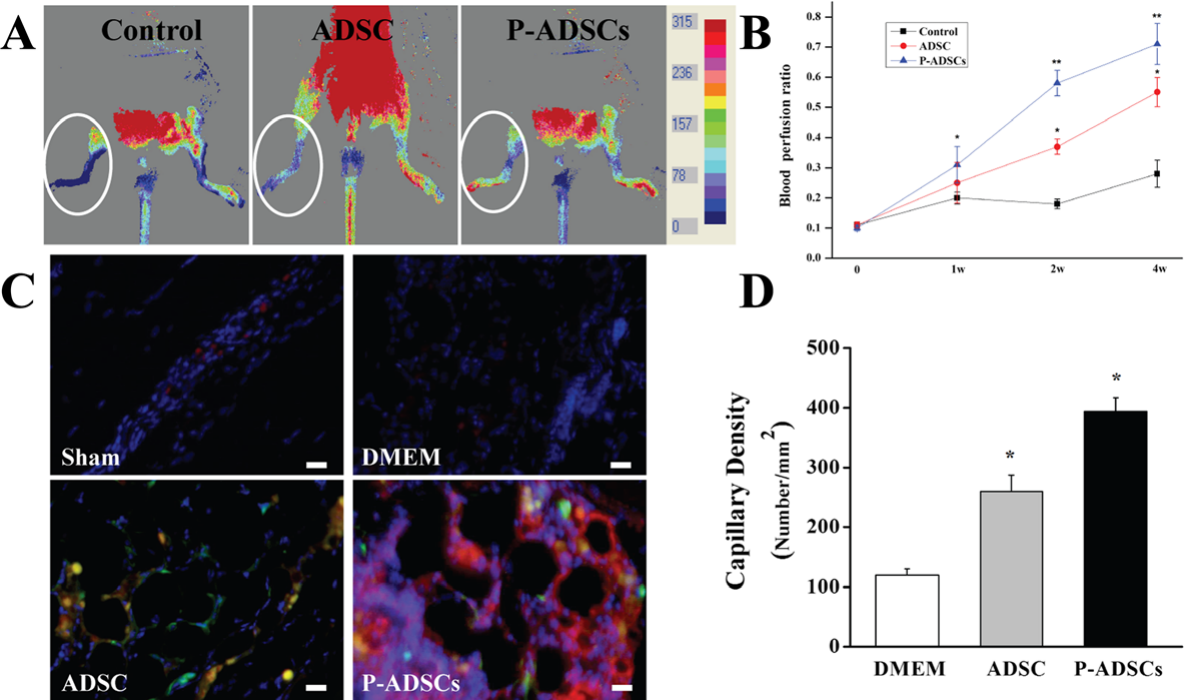
Effects of transplantation of P-ADSCs on blood flow and histology staining. Representative color laser Doppler images of superficial blood flow in lower ischemic limbs were taken 7, 14, and 28 days after transplantation **a**. The blood perfusion index was significantly higher in the P-ADSC group than those in ADSCs and DMEM control groups over 4 weeks **b**. **c** Representative fluorescent staining for CD31 (*red*), ADSCs (*green*), and nuclei with DAPI (*blue*) in the sham, DMEM, ADSC and P-ADSC groups. **d** Quantitative analysis indicated that the microvessel densities were significantly higher in the P-ADSC and ADSC groups than those in DMEM control groups (*P* <0.05). Scale bar = 100 μm. *P <0.05, **P <0.01. *ADSC* adipose-derived stem cell, *DMEM* Dulbecco’s modified Eagle’s medium, *P-ADSC* periostin-transfected ADSC

We evaluated the efficacies of periostin on ADSCs in Apo E-deficient mice with hind limb ischemia. Representative color laser Doppler images of superficial blood flow in lower ischemic limbs were taken 7, 14, and 28 days after transplantation. We found that the laser Doppler perfusion index was significantly higher in the P-ADSC group and ADSC group than that in the DMEM control group over 4 weeks (Fig. 6a, b).

**Fig. 7:**
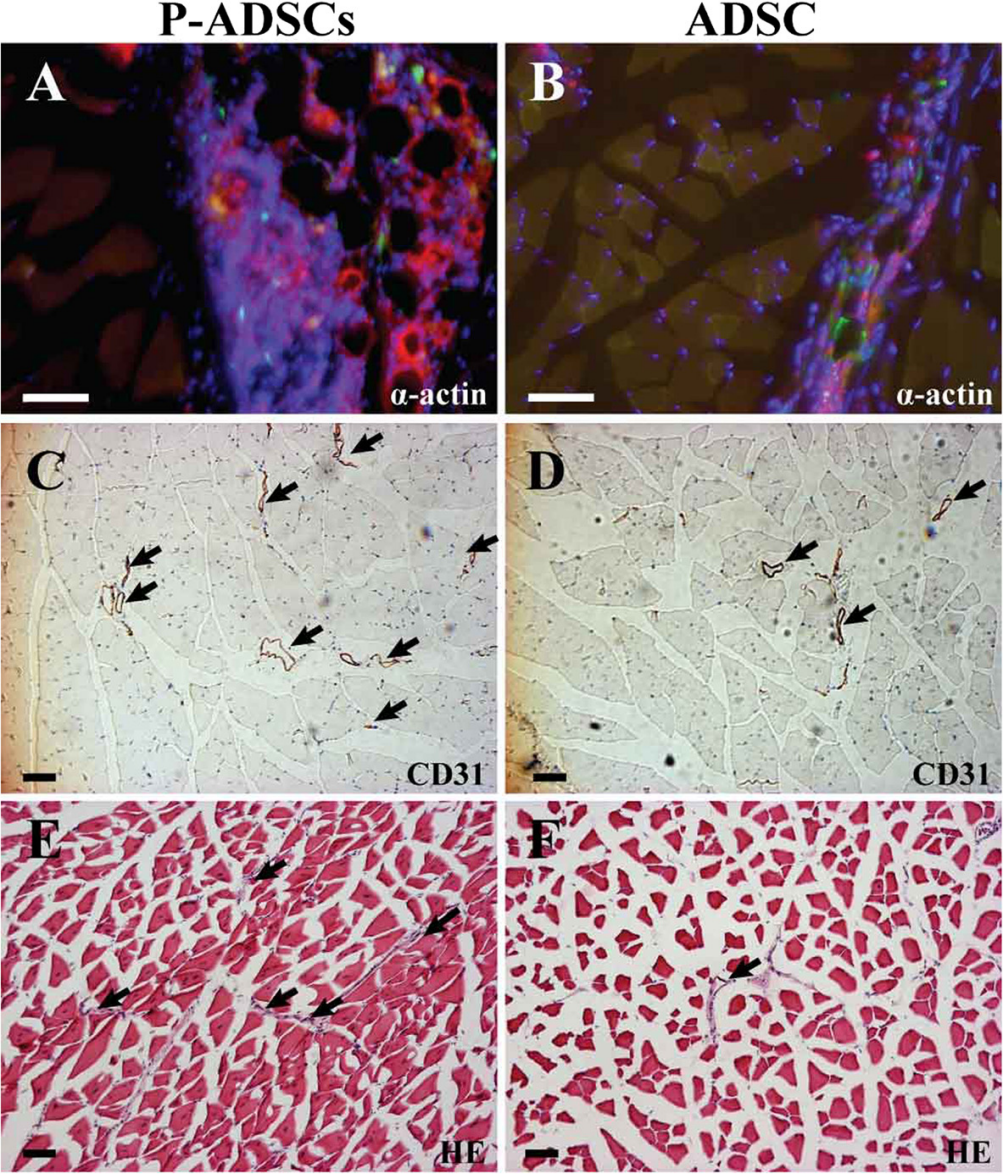
Histology staining of the ischemic muscles after cell transplantation. Representative fluorescent staining for α-actin (*red*), ADSCs (*green*), and nuclei with DAPI (*blue*) in the P-ADSC group **a** and in the ADSC group **b**. Representative immunohistochemistry staining for endothelial cell markers CD31 (*black arrows*) in the P-ADSC group **c** and in the ADSC group **d**. Representative HE staining in the P-ADSC group **e** and in the ADSC group **f** (*black arrows*, blood vessels). Scale bar = 100 μm. *ADSC* adipose-derived stem cell, *P-ADSC* periostin-transfected ADSC

No tumor or teratoma formation formed in the ischemic muscles and other organs after cell transplantation. Fluorescent microscopic examination detected GFP-expressed cells in the ischemic limb sites. The cells gradually migrated toward the ischemic areas over time and differentiated into the endothelium, as confirmed by CD31 (red) and GFP (green) double staining (Fig. 6c). These results indicated that the P-ADSCs migrated to the damaged area and participated in the repair of injured muscles. Quantitative analysis showed that the microvessel densities of the P-ADSCs were significantly higher than in the ADSC and DMEM control groups (*P* <0.05) (Fig. 6d). In addition, fluorescent staining for α-actin and immunohistochemistry staining for endothelial cell marker CD31 (black arrows) and quantification of small artery density showed that arterioles were significantly higher in the P-ADSC group (Fig. 7a, c) than in the ADSC group (Fig. 7b, d) (*P* <0.05; Additional file 2). HE staining indicated that new blood vessels were distributed between the muscle bundles and the number of capillaries by p-ADSC treatment (Fig. 7e, black arrows) was much greater when compared with ADSC-alone treatment (Fig. 7f, black arrows) (*P* <0.05, data not presented). Our results demonstrated that periostin-mediated adhesion is necessary for ADSC survival and differentiation.

### Cytokines and growth factors in P-ADSCs

**Fig. 8:**
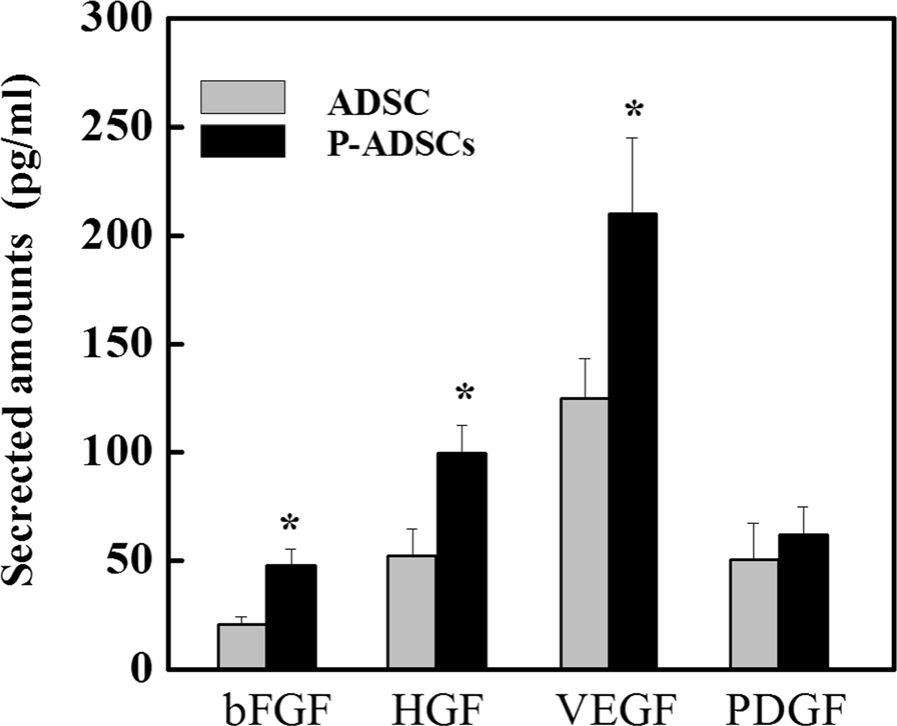
Cytokines and growth factors in P-ADSCs. ELISA showed that cytokines and growth factors including bFGF, HGF, and VEGF, but not PDGF, were upregulated in P-ADSCs compared with ADSCs. *P <0.05. *ADSC* adipose-derived stem cell, *bFGF* basic fibroblast growth factor, *HGF* hepatocyte growth factor, *P-ADSC* periostin-transfected ADSC, *PDGF* platelet-derived growth factor, *VEGF* vascular endothelial growth factor

To detect paracrine factors, we collected the supernatant of transfected or untransfected ADSCs at 48 hours for ELISA. Cytokines and growth factors, including bFGF, HGF, and VEGF, but not PDGF, was upregulated in P-ADSCs compared with ADSCs (Fig. 8). Our findings indicate that periostin stimulated the paracrine effects of ADSCs to promote ischemic muscle vascularization.

## Discussion

In this study, we acquired ADSCs from adipose tissues and adopted gene transfection to efficiently yield P-ADSCs. Periostin enhanced the viability, proliferation, and migration of ADSCs, and hypoxia-induced apoptosis in ADSCs was inhibited. Moreover, P-ADSCs were implanted into Apo E-deficient mice with hind limb ischemia. The laser Doppler perfusion index was significantly higher in the P-ADSC and ADSC groups compared with that in the control DMEM group. Immunofluorescence staining for 4 weeks indicated that the P-ADSCs were in and around the ischemic sites. Some cells differentiated into capillaries and endothelium. The improved microvessel density was directly attributed to the presence of P-ADSCs in the ischemic areas compared with the ADSC and DMEM groups. The associated upregulation of the integrin-β1/FAK/PI3K/Akt/eNOS signal pathway elucidated the possible molecular mechanism of the regulatory function of periostin in survival, migration, and vascularization of ADSCs.

Previous studies have reported that adipose tissues contain ADSCs, which are multipotent stem cells and can differentiate into numerous cell types and repair damaged tissues or organs [31–33]. However, the therapeutic efficiency of ADSCs was limited despite engrafting large numbers of stem cells. The lack of adhesion is probably the main cause of the low survival rate, adhesion, and migration following the transplantation of stem cells [28]. Effective strategies must be implemented to enhance the survival, adhesion, and migration ability of stem cells in the targeted areas. Several studies have documented that cell adhesion to the ECM is regulated by integrins that control biological processes such as gene expression, cell survival, proliferation, and differentiation [34, 35]. Periostin, one of the ECM, is highly expressed in tissue trauma or wound repair, and participates in the repair of tissue damage through direct interaction with other ECM proteins such as collagen, tenascin-C, and fibronectin [36, 37].

Our in-vitro study confirmed the function of periostin on the viability, adhesion, and migration of ADSCs, as well as determined the molecular mechanism on cellular signals. Lentiviruses, which were frequently used for modification, were used to transfect the periostin gene into ADSCs. Immunofluorescence staining revealed that more than 80 % of the ADSCs expressed periostin markers. Western blot analysis likewise showed that the periostin was overexpressed in transfected ADSCs compared with the control groups. Periostin enhanced the viability, and proliferation of ADSCs as well as inhibited the apoptosis of the cells under hypoxic conditions. In addition, western blot analysis revealed that the levels of anti-apoptotic Bcl-2 increased and the pro-apoptotic Bax decreased significantly. The apoptosis-related genes Bcl-2/Bax ratio was increased in P-ADSCs. Periostin has been reported to inhibit the apoptosis through the JNK signal pathway under hypoxic conditions [13, 36]. We observed that periostin promoted the adhesion and migration of ADSCs. In addition, western blot analysis revealed that phosphorylation of the focal adhesion-related kinases (e.g., FAK, PI3K, eNOS, and Akt) was significantly upregulated in the P-ADSC groups compared with the ADSC and siRNA groups, which exhibit strong ability to enhance cell proliferation, migration, and even survival [36]. In addition, we observed that integrin-β1 was also significantly increased, which is a major signal mediator of cell adhesion and migration. Periostin was reported to connect to several integrin subtypes (such as αvβ3, β1, β3, α4β6, and αvβ5) and also with focal adhesion kinases, which function as receptors that stimulate the downstream signaling pathways (such as PI3K/Akt/eNOS, etc.) which are playing a pivotal role in cell adhesion, survival, migration, and differentiation [36, 38, 39]. Kuhn et al. [19] confirmed that periostin promote the differentiation of cardiomyocytes by activating the myocardial cell surface αv, β1, β3, and β5 integrins. The integrin/FAK/Akt/PI3K/eNOS signaling pathway is essential for cell survival, proliferation, adhesion, and migration [39].

Our in-vivo immunofluorescence and histological staining indicated that P-ADSCs were in and around the ischemic muscle sites, with an increase in capillary density. The increase in microvessel density was closely bound with the laser Doppler perfusion index, which implies the improvement in hind limb function. Recent studies have showed that integrin-regulated adhesion is necessary for cell survival and vital for the differentiation of stem cells [36, 37]. Our results demonstrated that high expression of periostin in ADSCs considerably stimulated the expression of multiple growth factors (e.g., VEGF, HGF, bFGF, and PDGF), which alleviated the pathological hind limb ischemia muscle remodeling and promoted vascularization. Shao and Guo [40] demonstrated that periostin stimulated the high expression of VEGF receptor Flk-1/KDR in endothelial cells via the integrin-FAK-mediated signaling pathway. Although the molecular mechanisms of the therapeutic effects of ADSCs for angiogenesis remained complex, we demonstrated that periostin could enhance the survival, migration, and therapeutic efficiency of ADSCs. The results of this study yield novel insights into the treatment of critical limb ischemia.

## Conclusion

This study has demonstrated the critical role of periostin in protecting ADSCs from apoptosis, enhancement of survival of ADSCs, as well as adhesion and migration in vitro. Following the transplantation into the ischemic hind limb of the Apo E-deficient mice, P-ADSCs enhanced the laser Doppler perfusion index of the hind limb ischemia as well as promoted vascularization. The mechanisms underlying the salutary effects of P-ADSCs were associated with upregulation of the integrin-β1/FAK/Akt/PI3K/eNOS signaling pathway and the secretion of growth factors. Our results suggest that periostin is a potential therapeutic candidate for treating ischemic disease with gene-based stem cell therapy.

## Additional files

Additional file 1:
**is Figure S1 showing that P3 GFP-ADSCs were strongly double positive for GFP and the stem cell surface antigens CD44 and CD105, and negative for CD11b, CD31, CD34, CD45, CD133, and MHC-II.** (TIFF 2302 kb) Additional file 2:
**is Figure S2 showing that quantitative analysis indicated that the microvessel densities were significantly higher in P-ADSC and ADSC groups than those in DMEM control groups (*** ***P <*** **0.05).** (TIFF 68 kb)

## Abbreviations

ADSC: Adipose-derived stem cell
Akt: Protein kinase B
Apo E: Apolipoprotein E
bFGF: Basic fibroblast growth factor
CCK-8: Cell counting kit-8
DAPI: 4′,6-Diamidino-2-phenylindole
DMEM: Dulbecco’s modified Eagle’s medium
ECM: Extracellular matrix proteins
ELISA: Enzyme-linked innunosorbent assay
eNOS: Endothelial nitric oxide syntase
FBS: Fetal bovine serum
FAK: Focal Adhesion Kinase
GFP: Green fluorescent protein
HE: Hematoxylin and eosin
HGF: Hepatocyte growth factor
MHC: Major histocompatibility complex
OD: Optical density
P3: Passage 3
P6: Passage 6
P-ADSC: Periostin-transfected adipose-derived stem cell
PBS: Phosphate-buffered solution
PDGF: Platelet-derived growth factor
PE: Phycoerythrin
PET: Polyethylene terephthalate
PI3K: Phosphoinositide-3-kinase
qRT: Quantitative real-time
SD: Standard deviation
siRNA: Small interfering RNA
VEGF: Vascular endothelial growth factor
