# Supplementary figures and images for: Periostin enhances adipose-derived stem cell adhesion, migration, and therapeutic efficiency in Apo E deficient mice with hind limb ischemia
Source: Stem Cell Res Ther. 2015 Jul 24;6(1):138. doi: 10.1186/s13287-015-0126-x (PMC4533765; doi:10.1186/s13287-015-0126-x)

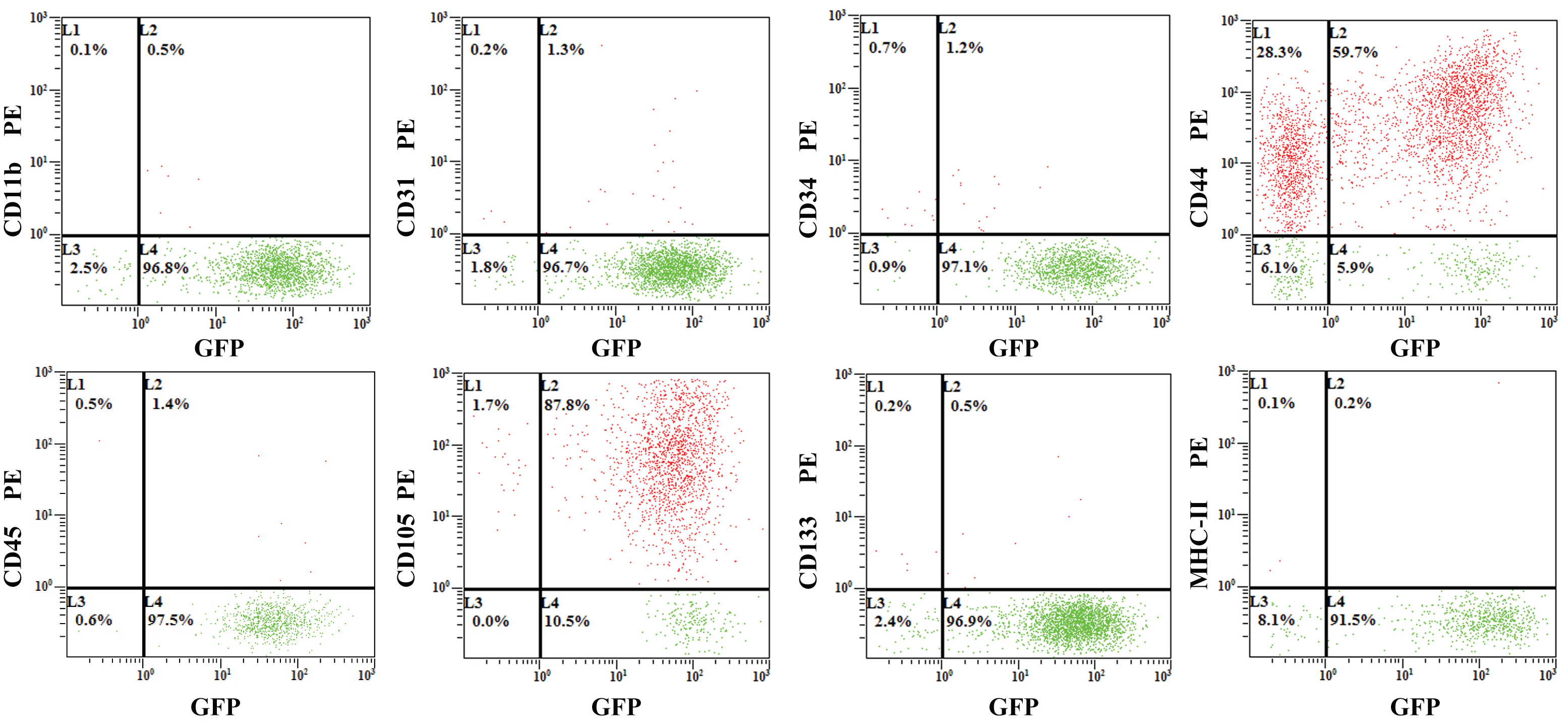

Supplement: Additional file 1: — is Figure S1 showing that P3 GFP-ADSCs were strongly double positive for GFP and the stem cell surface antigens CD44 and CD105, and negative for CD11b, CD31, CD34, CD45, CD133, and MHC-II. (TIFF 2302 kb) [file 13287_2015_126_MOESM1_ESM.tiff]

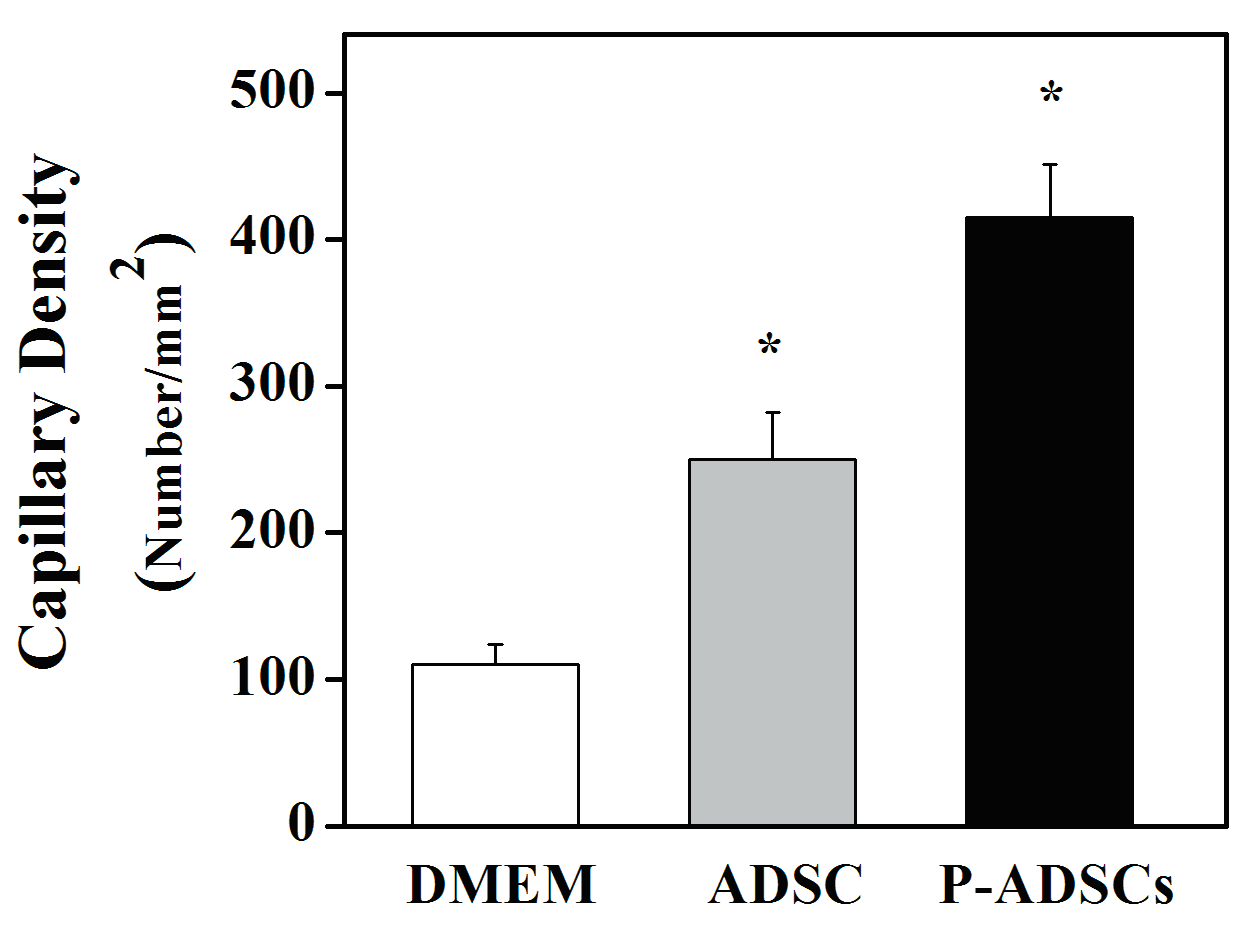

Supplement: Additional file 2: — is Figure S2 showing that quantitative analysis indicated that the microvessel densities were significantly higher in P-ADSC and ADSC groups than those in DMEM control groups (* P < 0.05). (TIFF 68 kb) [file 13287_2015_126_MOESM2_ESM.tiff]
